# How Do Upper Echelons Perceive Porter’s Five Forces? Evidence From Strategic Entrepreneurship in China

**DOI:** 10.3389/fpsyg.2021.649574

**Published:** 2021-12-06

**Authors:** Chengqi Shi, Comfort Afi Agbaku, Fan Zhang

**Affiliations:** School of Economics and Management, Shaanxi University of Science and Technology, Xi’an, China

**Keywords:** strategy, entrepreneurship, exploratory factor analysis (EFA), Porter’s five forces model, China

## Abstract

Porter’s five forces model is an authoritative management tool used in analyzing the profitability and attractiveness of industries through an outside-in viewpoint. In the past decade, dramatic and rapid changes have prompted some criticism of the model. The comparison between new and old economy analysis makes the fundamentals of the model seem weak. Moreover, the past decade has shown that strategy and entrepreneurship in China are not completely dependent on the model. This study first aims to verify the sustainability of the five forces model and analyze its integration into China’s entrepreneurial economy. By conducting in-depth interviews among the upper echelons from various industries, it was found that along with the competitive factors emphasized by the model, Chinese entrepreneurs attend to cooperative factors such as Guanxi, the Chinese term for relationship, and the possibilities of technology integration with the five forces. They also tend to enlarge the strategic view to consider factors such as how the market evaluates the forces. To verify these findings, the authors carried out a large-scale survey with a modified questionnaire analyzing the data collected using exploratory factor analysis with SPSS 22. The outcome shows that Porter’s model is still valid to some extent. Companies are still working in a network of buyers, suppliers, substitutes, new entrants, and competitors. However, reinventions are necessary to include the new factors of Guanxi, technology (e-commerce and logistics), and marketing and branding, which have changed the structure of the industry. These factors arise from the cooperative nature of Chinese culture and may have equal or even larger significance compared with their competitive counterparts in today’s business world.

## Introduction

The goal of every business is to achieve its objectives or targets effectively. Can these objectives be obtained without much attention to the strategic forces that drive business? When asked what forces are most important to entrepreneurship and strategy in the business world, any scholar, entrepreneur, or business executive in the western world will refer to Porter’s five forces model. The basic aim of this model is to describe the competitive environment of firms in terms of five industry-specific factors (Porter, 2008).

In recent years, these forces have been questioned, as researchers argue that the model has innate weaknesses and is difficult to operationalize (Lee et al., 2012). It does not take into account a firm’s potential relations with the determinants of the industry environment (Dulčić et al., 2012). There is a need to know the limitations of the model and develop a new one to reflect and direct the new business world, which has changed fundamentally since the 1970s. In addition to the criticisms from western academia, entrepreneurial growth in China has increased in recent times. This entrepreneurial growth has brought many changes to China and the world. Wang and Chang (2009) argue that Chinese culture differs from western culture. They challenged the five forces model and came up with a new model with five new forces: business purpose, business climate, business location, business leader, and business organization. Although they provide no statistical analyses to support their argument, their research is innovative and worthy of consideration.

China is setting high standards in today’s businesses and entrepreneurial ventures. It has become a major player in the business world. The study on the sustainability of Porter’s five forces model and its possible improvement in the Chinese context is worthwhile in investigating the success of the Chinese economy. Such a study may benefit other economies aiming to compete or cooperate with China. Therefore, this study aims to resolve the following research questions: Does Porter’s five forces model impact entrepreneurial strategic decisions in China? What strategic forces drive entrepreneurial strategic decision-making in China, and how do they compare with Porter’s model?

This study addresses these questions by examining the sustainability of Porter’s five forces model in Chinese businesses to investigate if the framework is used to ascertain profitability and attractiveness in various industries. If this is not the case, what are the strategic forces that are impacting these sectors? The study’s objectives focused on: (1) analyzing the extent to which Porter’s five forces model affects entrepreneurial strategic decisions in China, (2) finding out what strategic forces drive entrepreneurship and strategy in China, and (3) exploring the possibility of applying these new forces beyond the Chinese business context by constructing a more effective model.

## Research Background and Literature Review

Porter created the five forces model at the Harvard Business School in the 1970s. The model classifies five forces in a microenvironment that motivate competition and threaten a firm’s profit-making capacity. The forces are rivalry, consumers’ and suppliers’ negotiating powers, threat of newcomers, and alternatives. Porter’s five forces originated in the industrial economic approach. The concept was that the market structure could determine the attractiveness and overall profitability of a market (Slater and Olson, 2002). The market structure can impact a firm’s strategic behavior, and a competitive strategy could bring success. Therefore, the success of the organization is indirectly reliant on the market structure. According to Porter, “being aware of these forces helps a business hold its spot in the industry with little exposure to attack” (Porter, 2008, p. 137).

In western academia, and in the current business world, there is an ongoing debate on the sustainability of Porter’s model. Johnson et al. (2008) considered the framework to be a powerful device with an outside-in perspective identifying where power lies. Most businesses around the globe are trying to improve their skill set and increase their industry assets by optimizing the opportunities available in the market and managing the problems and challenges. The only way for them to achieve this is to know their working environment because this dynamic external and internal environment has many variables affecting the company and its market value. The five forces framework is a “useful starting point for strategic analysis even where profit criteria may not apply” (Johnson et al., 2008, p. 60). Nguyen (2017) conducted a case study in a small construction company and concluded that a firm can gain or lose competitive advantage depending on how well it applies diverse significant strategic analytical tools such as Porter’s five forces. Mugo (2020) also found that Porter’s five forces framework influenced the performance of telecommunication firms in Kenya.

The Monitor Group, a consulting company partnered with Porter, filed for insolvency in November 2012. Dennings (2012) linked the consulting firm’s problems to a conceptual mistake made by Porter: “In Porter’s theoretical landscape invention, all strategy worthy of the name involves avoiding competition and seeking out above-average profits protected by structural barriers. A strategy is wholly assumed as a method of making additional profits without planning to produce a better product or service.” Love (2013) argues that, in the long term, this method does not benefit society or the firm. Slater and Olson (2002) state that although Porter’s model is still as viable in this era as it was 20 years ago and there are not many changes to be made, there is a need to focus on new elements that were not fundamental in the five forces model and also to rethink the model. Andriotis (2004) believed that as time passes, Porter’s five forces may change, as will the comparative importance of the model. Mohapatra (2012) states that individual forces and their cooperative impact will change as the government policies and macroeconomic and environmental conditions change. Aktouf et al. (2005) analyzed the viability of a particular industry with Porter’s framework and realized that the model was obsolete and needed improvement. Dulčić et al. (2012) proposed three new forces that align well in today’s business context: digitization, globalization, and deregulation. Jaradat and Almomani (2013) recommended the need to take into consideration the implementation of the model by industrial food companies to enable them to select suitable business strategies effectively. The authors added the new factors of the impacts of the external and internal environments from the top management perspective. Shamir and Johnson (2014) provided an in-depth literature review of the latest findings on Porter’s competitive force model and concluded that the five competitive forces model could be partly rejected and that four additional forces could compensate for the model’s innate weakness: digitalization, globalization, deregulation, and level of innovativeness. McAran and Manwani (2016) proposed “context” as an additional important factor for the model, finding that the original forces were not equally significant in different industries. Isabelle et al. (2020) investigated the relevance of Porter’s framework through case studies and proposed a modified framework augmented by four additional forces: the competitors’ level of innovativeness, exposure to globalization, threat of digitization, and industry exposure to deregulation activities.

Today’s business world has witnessed significant changes. The Chinese economy, in particular, is booming globally, and China’s huge market is attractive to enterprises abroad (Tsui et al., 2017). As the world’s fastest-growing economy, China’s strategic experience in the last 10 years might serve as the best guide for the next 10 years. Business leaders should adopt a new Chinese strategy to be more successful. This strategy does not merely mean a set of plans for doing business in China. Most big companies are already doing business and competing with China, and the smaller firms will soon join them. The Chinese strategy is a changed one-world strategy with a long-term development plan to do business as a worldwide initiative with China at the center, playing a different role from the role played in the past (Tse, 2010). Entrepreneurship and innovation contribute greatly to China’s economic development. Innovation is a central activity for firms wishing to launch new ventures and to renew their firm’s strategic efforts (Ginsberg and Guth, 1990; Covin and Miles, 1999). Successful innovation is often complicated and requires firms to exhibit multiple talents and competencies. Strategic entrepreneurship is effective in the formation of business strategies involving simultaneous opportunity-seeking and advantage-seeking behaviors. Firms exhibiting a strong entrepreneurial orientation may have an advantage in undertaking innovation *via* exploration and exploitation (Dess, 2005). Entrepreneurial orientation is a strategy-making practice or process engaged in by managers to recognize and generate venture opportunities. Some firms link much of their success to entrepreneurial orientation (Scheela, 2001).

In short, it is uncertain whether Porter’s five forces are still sustainable. The framework was created in 1979 and has been available for more than 40 years without any alterations. The evidence from China indicates the need for in-depth analysis on the relevance of the framework and on possible ways to reinvent it in the business world of today.

## Methods and Data

The research was conducted in two stages. In the first stage, primary data was gathered to analyze whether industries should reexamine Porter’s forces through in-depth investigations and interviews with representatives of the upper echelons of many business sectors in China. The process started with a questionnaire with structured questions taken from a classic five forces analysis scale (Lee et al., 2012), with the addition of some open-ended questions such as “what are the strategic forces used to measure the competitiveness, attractiveness, and profitability of a certain industry or market in China?” A total of 22 entrepreneurs from various industries were interviewed to generate qualitative and quantitative data. Following analysis of the pretest data, the questions were gradually adjusted to reflect the responses of the pretest interviewees. At the end of the stage, the standard items were purified with Cronbach’s alpha coefficient and corrected item-total correlation. It was found that in addition to Porter’s five competitive factors, Chinese entrepreneurs pay considerable attention to cooperative factors such as Guanxi (i.e., relationship in Chinese, but having a broader meaning including interdependence, cooperation, and partnership) and the possibilities of integrating technology into the forces. They also tend to enlarge the strategic view and consider factors such as how the market evaluates the five forces. Therefore, these related items were added to the second-stage questionnaire.

In the second stage, a random sampling technique was conducted to investigate the applicability of Porter’s five forces model in China and to explore possible evidence-based revisions in the Chinese context. The final questionnaire was generated in two parts. The first part constituted background information. The second part surveyed entrepreneurs’ major concerns regarding the five forces when making strategies, including a 5-point Likert scale with 46 items, as shown in Table 1, and two open-ended questions. The questionnaires were sent to managers in various industries, and there were 156 valid responses. The data were analyzed with the software package SPSS 22 and exploratory factor analysis (EFA) to identify the impact of Porter’s five forces on Chinese businesses. Factor analysis is a multivariate method used for data reduction purposes. The basic idea of this technique is to represent a set of variables by a smaller number of variables (Johnson and Wichern, 1982). The method was mainly designed for interval data, although it can also be used for ordinal data (e.g., scores assigned to Likert scales). The variables utilized in factor analysis should be linearly associated with one another. The variables should be moderately correlated. If this is not the case, the number will be almost identical to the original variables.

**TABLE 1:** Structured items constituting the final questionnaire.

**Forces**	**Items**
New entrants	Customer switching costs*
	Initial capital requirement*
	Brand loyalty*
	Government regulation*
	Economies of scale*
	Cost advantages*
Substitutes	Number of substitutes*
	Closeness of substitutes*
	Buyer propensity to substitutes
	Other technologies*
	Conformity of substitutes to upstream and downstream technical standards
	The degree of technology integration between the substitutes and upstream and downstream
	The ready availability of substitutes and emergent of new ones
	The degree of close cooperation between substitutes and related industries
	Market awareness of alternative brands
	The relative price of substitutes
Buyers/ customers	Backward integration*
	Importance to buyers*
	Buyer switching costs*
	Dependence on buyer industry*
	Buyer portfolio*
	Product uniqueness*
	Close cooperation with buyer (frequency and years)
	Compliance with buyer’s technical standards
	Buyer information about demand, actual market price, and suppliers cost
	Market awareness of buyer’s brand
	Degree of technical integration with buyer
Suppliers	Supplier switching costs*
	Supplier portfolio (size and quantity, etc.)*
	Importance of suppliers*
	Forward integration*
	Dependence on supplier industry*
	Degree of technical integration with suppliers
	Close cooperation with suppliers (frequency and years)
	Supplier uniqueness
	Consistent with supplier’s technical standards
	Market awareness of supplier brands
The industry	Differentiation among companies*
	Industry demand and capacity*
	Industry structure*
	Exit barriers*
	The gap between technology and competitors
	Fraud by lawyers, employees, etc.
	Potential partnerships with competitors
	Existing partnerships with competitors
	Government regulation/policy

*Comes from a classic five forces analysis scale (Lee et al., 2012), others come from the pre-test interview in the Chinese context, as shown in the following tables.

Consider the observable random variable X with p-components, with mean μ, and covariance matrix Σ. The factor model postulates that X is linearly dependent on the few unobservable random variables *F*_1_, *F*_2_……., *F*_*m*_, called common factors, and additional sources of variation ε_1_, ε_2_,……., ε_*p*_, called specific factors. The generalized factor analysis model can be expressed as,


(1) $$X_{1}-\mu_{1}=l_{11}\,F_{1}+l_{12}\,F_{2}+\ldots \,\ldots \,\ldots \,\ldots .+l_{1\,m}\,F_{m}+\varepsilon_{1}$$

(2) $$X_{2}-\mu_{2}=l_{21}\,F_{1}+l_{22}\,F_{2}+\ldots \,\ldots \,\ldots \,\ldots .+l_{2\,m}\,F_{m}+\varepsilon_{2}$$

(3) $$X_{p}-\mu_{p}=l_{p\,1}\,F_{1}+l_{p\,2}\,F_{2}+\ldots \,\ldots \,\ldots \,\ldots .+l_{p\,m}\,F_{m}+\varepsilon_{p}$$

## Results and Analysis

This section contains an analysis of the results gathered from the online survey. The first part constitutes background information. The second part shows the industry’s awareness of Porter’s five forces and its impact on them. The challenge of gathering data required the responses to be divided into two parts: close-ended responses and open-ended responses.

### Background Information

Of the respondents, 26.9% indicated that they have been operating for over 20 years in their respective industries, which means that they have the adequate experience to know what or which type of strategic management to apply in the operations of their business to enforce competitive advantage (as shown in Table 2). Therefore, they should be well informed as to whether Porter’s five forces model has any impact on assessing the attractiveness of a business and on equipping them to gain a competitive advantage.

**TABLE 2:** Number of years in operation.

**Number of years in operation**	**1–5**	**6–10**	**11–15**	**16–20**	**Over 20**
Percentage	28.8%	19.2%	13.5%	11.5%	26.9%

Of the respondents, 100% indicated whether the business in China is local-owned, foreign-owned, or both local- and foreign-owned. These data show the influence of western companies on Chinese trade and business practices. Table 3 shows that 82.7% of the businesses that took part in this survey were locally-owned and that 5.8% were foreign-owned, making it somewhat difficult for Porter’s forces to influence how these businesses are run. Nevertheless, 11.5% of the companies were also local and foreign-owned, representing not an insignificant percentage.

**TABLE 3:** Type of ownership.

**Type of ownership**	**Local-owned**	**Foreign-owned**	**Both owned**
Percentage	82.7%	5.8%	11.5%

### Information on Porter’s Five Forces Model

#### New Entrants

##### Factor Analysis

The primary data were measured on a 5-point Likert scale with the following choices: very high, reasonably high, average, relatively low, and very low. Kaiser–Meyer–Olkin (KMO) and Bartlett’s test were conducted before factor analysis. The KMO index generally ranged from 0 to 1 (Williams et al., 2010). According to Frohlich and Westbrook (2001), a minimum KMO score of 0.50 is considered enough for factor analysis. A large KMO value (0.782) and Bartlett’s test of sphericity (0.000) indicate that data collected for factor analysis is adequate. A principal component factor analysis with Varimax rotation was carried out for items indicating the threat of new entrants affecting organizations. The eigenvalue for the factor is 2.948, which cumulatively explains 49.141% of the total variance for the threat of new entrants (Table 4).

**TABLE 4:** Total variance explained: New entrants.

**Component**	**Initial Eigenvalues**			**Extraction Sums of Squared Loadings**		
	**Total**	**% of Variance**	**Cumulative%**	**Total**	**% of variance**	**Cumulative%**
1	2.948	49.141	49.141	2.948	49.141	49.141
2	0.811	13.513	62.654			
3	0.734	12.229	74.883			
4	0.618	10.308	85.190			
5	0.576	9.600	94.790			
6	0.313	5.210	100.00			

##### Analysis of Factors

Table 5 shows the factor loadings of the correlation coefficient of factors influencing the threat of new entrants by using principal component analysis (PCA). This factor has a significant factor loading on these variables that have formed an influential group pertaining to economies of scale, government regulation, brand loyalty, cost advantages, initial capital requirement, and customer switching costs. Therefore, it can be said that these factors provide a basis for affecting the threat of new entrants into the Chinese market.

**TABLE 5:** Factors loadings of correlation coefficient: New entrants.

	**Component**
	**Factor 1**
Customer switching costs*	0.814
Initial capital requirement*	0.744
Brand loyalty*	0.701
Government regulation*	0.664
Economies of scale*	0.658
Cost advantages*	0.606

#### Substitutes

##### Factor Analysis

A large value KMO value (0.825) and Bartlett’s test of sphericity (0.000) indicate that the data are adequate (reliable) for the application of factor analysis. The findings show that a relationship exists between the variables at (*p* < 0.05.). The eigenvalues for the factors are 4.745 and 1.289, showing 60.340% of the total variance for the determinants of the threat of substitutes, as shown in Table 6.

**TABLE 6:** Total variance explained: Substitutes.

**Component**	**Initial Eigenvalues**		**Extraction Sums of Squared Loadings**		**Rotation Sums of Squared Loadings**	
**Total**	**% of Variance**	**Cumulative%Total**	**% of Variance**	**Cumulative%Total**	**% of Variance**	**Cumulative%**
14.745	47.449	47.4494.745	47.449	47.4493.318	33.177	33.177
21.289	12.892	60.3401.289	12.892	60.3402.716	27.163	60.340
30.809	8.094	68.434				
40.695	6.948	75.382				
50.636	6.364	81.746				
60.573	5.730	87.476				
70.471	4.711	92.187				
80.320	3.205	95.392				
90.259	2.591	97.983				
100.202	2.017	100.000				

##### Analysis of Factors

Table 7 shows the factor loadings of the correlation coefficient of factors influencing the threat of substitutes by using PCA. The elements of Factor 1 are conformity of substitutes to upstream and downstream technical standards, the degree of technology integration between the substitutes and upstream and downstream, the ready availability of substitutes and the emergence of new ones, the degree of close cooperation between substitutes and related industries, market awareness of alternative brands, and the relative price of substitutes. The elements of Factor 2 are the number of substitutes, closeness of substitutes, and buyer propensity to buy substitutes and other technologies. Factor 1 values the interdependent relationship between the focus and its substitutes, such as potential for cooperation, technology integration, and market context. Factor 2 mainly emphasizes on traditional competition factors.

**TABLE 7:** Factors loadings of correlation coefficient: Substitutes.

	**Components**	
	**Factor 1**	**Factor 2**
Conformity of substitutes to upstream and downstream technical standards	0.828	0.165
The degree of technology integration between the substitutes and upstream and downstream	0.823	−0.019
The ready availability of substitutes and emergent of new ones	0.687	0.378
The degree of close cooperation between substitutes and related industries	0.682	0.317
Market awareness of alternative brands	0.638	0.341
The relative price of substitutes	0.529	0.482
Number of substitutes*	0.161	0.867
Closeness of substitutes*	0.103	0.835
Buyer propensity to substitutes	0.303	0.659
Other technologies*	0.449	0.460

#### Buyers/customers

##### Factor Analysis

The KMO value is 0.799, far greater than the necessary factoring value of 0.5. The significant value for Bartlett’s test was *p* = 0.000 with a Chi-square value of 947.350 and DF = 55, thereby verifying that the item has been factored in. The eigenvalue for the factor is 6.086, representing 62.136% of the total variance for the determinants of the bargaining power of buyers/customers (Table 8).

**TABLE 8:** Total variance explained: Buyers/Customers.

**Component**	**Initial Eigenvalues**		**Extraction Sums of Squared Loadings**		**Rotation Sums of Squared Loadings**	
**Total**	**% of Variance**	**Cumulative%Total**	**% of Variance**	**Cumulative%Total**	**% of Variance**	**Cumulative%**
15.649	51.350	51.3505.649	51.350	51.3503.962	36.017	36.017
21.186	10.786	62.1361.186	10.786	62.1362.873	26.119	62.136
30.891	8.102	70.238				
40.706	6.419	76.657				
50.606	5.508	82.166				
60.485	4.408	86.574				
70.463	4.211	90.785				
80.320	2.911	93.696				
90.312	2.834	96.530				
100.256	2.324	98.854				
110.126	1.146	100.000				

##### Analysis of Factors

Table 9 demonstrates the factor loadings of correlation coefficients based on the Varimax rotation of factors influencing the determinants of the bargaining power of buyers/customers by using PCA. The study results specify that two elements account for 62.136% of the total variance, as shown in Table 10. The elements of Factor 1 are close cooperation with the buyer (frequency and number of years), buyer information about demand, actual market price, and suppliers cost, backward integration, compliance with buyer’s technical standards, importance to buyers, market awareness of buyer’s brand, and degree of technical integration with the buyer. The elements of Factor 2 are buyer switching costs, dependence on buyer industry, buyer portfolio, and product uniqueness. Factor 1 centers on the interdependent relationship between the focus and its buyers/customers, whereas Factor 2 mainly emphasizes traditional competition factors.

**TABLE 9:** Component matrix with rotation varimax and factor loading: Buyers/Customers.

	**Components**	
	**Factor 1**	**Factor 2**
Close cooperation with buyer (frequency and years)	0.859	−0.010
Buyer information about demand, actual market price, and suppliers cost	0.719	0.342
Backward integration*	0.709	0.219
Compliance with buyer’s technical standards	0.689	0.369
Importance to buyers*	0.678	0.296
Market awareness of buyer’s brand	0.674	0.363
Degree of technical integration with buyer	0.648	0.448
Buyer switching costs*	0.215	0.850
Dependence on buyer industry*	0.157	0.798
Buyer portfolio*	0.329	0.742
Product uniqueness*	0.466	0.493

**TABLE 10:** Total variance explained: Suppliers.

**Component**	**Initial Eigenvalues**			**Extraction Sums of Squared Loadings**		
	**Total**	**% of Variance**	**Cumulative%**	**Total**	**% of Variance**	**Cumulative%**
1	5.845	58.445	58.445	5.845	58.445	58.445
2	0.836	8.360	66.805			
3	0.732	7.321	74.126			
4	0.694	6.941	81.068			
5	0.468	4.677	85.744			
6	0.419	4.189	89.933			
7	0.367	3.666	93.599			
8	0.309	3.089	96.687			
9	0.184	1.839	98.527			
10	0.147	1.473	100.000			

#### Suppliers

##### Factor Analysis

The KMO value is 0.858, indicating that the data are satisfactory for the application of EFA. The findings indicate that a relationship exists between the variables at a *p*-value of less than 0.05. In addition, the estimated eigenvalue for the factor is 5.845, which explains the 58.445% total variance for the significant determinants of the supplier’s power, as shown in Table 10.

##### Analysis of Factors

This factor also has significant factor loading that ranges from moderate to high on these variables, forming a critical cluster (Table 11). The elements include supplier switching costs, supplier portfolio (size and quantity), degree of technical integration with suppliers, close cooperation with suppliers (frequency and years), supplier uniqueness, importance of suppliers, consistency of supplier’s technical standards, market awareness of supplier brands, forward integration, and dependence on the supplier industry. Hence, it can be said that the following factors are considered as the significant determinants of a supplier’s power, among which the new factors from the Chinese context have relatively high explanatory power.

**TABLE 11:** Factors loadings of correlation coefficient: Suppliers.

	**Component**
	**Factor 1**
Supplier switching costs*	0.847
Supplier portfolio (size and quantity, etc.)*	0.833
Degree of technical integration with suppliers	0.817
Close cooperation with suppliers (frequency and years)	0.805
Supplier uniqueness	0.749
Importance of suppliers*	0.747
Consistent with supplier’s technical standards	0.745
Market awareness of supplier brands	0.714
Forward integration*	0.707
Dependence on supplier industry*	0.657

#### The Industry

##### Factor Analysis

The value of KMO is 0.809, higher than the necessary factoring value of 0.5. The significant value for Bartlett’s test was *p* = 0.000 with a Chi-square value of 654.601, and DF = 36 indicates that the item has been factored. The calculated eigenvalues for the factors are 4.452 and 1.071, representing 61.364% of the total variance for the determinants of competition, as shown in Table 12.

**TABLE 12:** Total variance explained: The industry.

**Component**	**Initial Eigenvalues**		**Extraction Sums of Squared Loadings**		**Rotation Sums of Squared Loadings**	
**Total**	**% of Variance**	**Cumulative%Total**	**% of Variance**	**Cumulative%Total**	**% of Variance**	**Cumulative%**
14.452	49.469	49.4694.452	49.469	49.4692.778	30.866	30.866
21.071	11.896	61.3641.071	11.896	61.3642.745	30.498	61.364
30.915	10.166	71.531				
40.690	7.666	79.197				
50.596	6.624	85.821				
60.465	5.162	90.983				
70.376	4.177	95.160				
80.291	3.238	98.398				
90.144	1.602	100.000				

##### Analysis of Factors

Table 13 shows the factor loadings of the correlation coefficient of factors influencing the rivalry among existing competitors by using PCA. The factor includes the major influential determinants of competition in the Chinese industry. It consists of items of government regulation/policy, industry demand and capacity, industry structure, the gap between technology and competitors, and differentiation among companies, emphasizing the macro dimension of the industry environment. Factor 2 includes fraud by lawyers, employees, or others; potential partnerships with competitors; existing partnerships with competitors; and exit barriers. It tends to indicate the microdimension of the industry environment.

**TABLE 13:** Component matrix with rotation varimax and factor loading: The industry.

	**Components**	
	**Factor 1**	**Factor 2**
Government regulation/policy	0.803	0.102
Industry demand and capacity*	0.790	0.201
Industry structure*	0.642	0.325
The gap between technology and competitors	0.603	0.332
Differentiation among companies*	0.602	0.372
Fraud by lawyers, employees, etc.	0.154	0.926
Potential partnerships with competitors	0.254	0.764
Existing partnerships with competitors	0.294	0.736
Exit barriers*	0.442	0.597

This article aims to determine if Porter’s five forces model is practiced in China. Of those surveyed, 78.2% agreed that Porter’s five forces are used to measure competition intensity, attractiveness, and profitability, whereas 21.8% of those surveyed disagreed. This outcome has a significant impact on our findings.

The respondents were also asked open-ended questions, and their responses were essential in building the forces linked to Porter’s five forces model.

(a) The respondents were asked what recommendations they would consider to make their industry more attractive. Some respondents stated that the firms should concentrate on putting in place a high-quality management system and an efficient resource allocation. They thought that firms should take more responsibility for having an attractive industry. Others focused on innovation, such as increasing the level of digital construction, providing customized services, and expanding into new markets as they broaden the channels for profit. Finally, some respondents indicated that optimal service and high product quality could give a company a competitive advantage.
(b) The respondents were also asked if they thought that Porter’s five forces, as listed above, are used to measure competition intensity, attractiveness, and profitability of an industry or market in China. If this is not the case, then what do they think the strategic forces are used to measure. Of the respondents, 21.8% indicated that Porter’s forces are not used to measure competition intensity, attractiveness, and profitability. However, some strategies use quality service, innovation, good relationships, good government policy, good after-sales service, e-commerce, and attending to market trends.

## Conclusion

### Findings

Based on the investigation, this article concludes that Porter’s five forces model impacts entrepreneurial strategic decisions in China. Most of the managers’ responses showed that they are well aware of the forces. The respondents also acknowledged that the model was used to check industry profitability and attractiveness during startups and expansions. However, gaps still exist between Chinese business practice and the classic western analysis model. Among the most frequently mentioned strategic forces relating to the drivers of decision-making in China, the top ones are good relationship, e-commerce, innovation, and quality services, rather than competition. The core of Porter’s five forces is derived from Darwinism in business, which assumes that the environment is composed of predetermined constants and that firms compete with each other for limited resources. Therefore, outsiders are threats. However, the Chinese business philosophy is different. Chinese entrepreneurs believe that the industry is an ecosystem, and all players within the industry, such as the buyers, suppliers, competitors, substitutes, and new entrants, are interdependent and evolve together. There are no permanent enemies. Competition is temporary because firms need to cooperate to develop and sustain a convenient business environment. Therefore, their strategic focus covers competition and cooperation, and their entrepreneurial view goes beyond individual firms to the whole system, which is eventually evaluated by the market.

**FIGURE 1:**
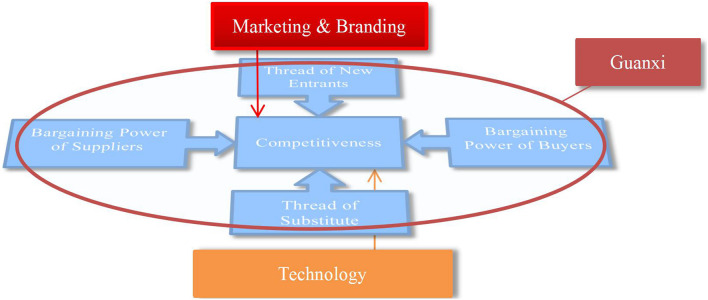
A reinvented competitive forces model for industries.

Based on the above, the authors created a more structured strategic model. Before proposing this reinvented model of competitive forces, it should be stressed that the primary notions of Porter’s five forces are as valid today as they were 40 years ago. As a result, it is not the intention to challenge the points Porter made so successfully. This article concentrates on the forces used in China’s fast-moving entrepreneurial and innovative economy, linking them with the original model, giving the western world an idea of what motivates Chinese entrepreneurs. In this respect, Figure 1 symbolizes a reinvented competitive forces model, which reconfigures Porter’s five original forces by adding three additional forces, specifically the concept of Guanxi, technology, and marketing and branding. These three forces have been added because, in the above EFA results, almost every item related to these concepts has a high factor loading. Some new factors emphasizing interdependence are found in evaluating the traditional five forces. These concepts come from a totally different business culture, but they can merge well with the model. They fit in the same framework, adding more comprehensive and long-term considerations when evaluating a firm’s industry environment, such as existing and potential relationships, technology compatibility, and brand influence among the players. While the original Porter’s five forces model was of value, the inclusion of the Chinese forces makes the model much more useful in helping firms to gain a competitive advantage.

#### Guanxi

In China, the concept of relationship is of the utmost importance. Personal information is often sought while business is being conducted or a business relationship with potential clients is being nurtured. Information on the net worth of an individual or their partner’s eastern astrology symbol is often shared. In this context, it is much more important to cooperate than to compete. Therefore, when doing business in China, one should concentrate on building a strong relationship.

#### Technology

Chinese upper echelons attach importance to technology innovation, to building technological platforms with cooperators, and to adopting dominant technical standards in the industry. This outlook holds true in today’s e-world. The advancement of technology has transformed China’s e-commerce. China has overtaken the United States and the EU in real online revenues with a vast improvement in its infrastructure and boundless sales. China’s online retail sales expanded rapidly over the past decade and retained a year-on-year growth of 27.3%, which is above the average growth rate worldwide. In 2019, the country’s share of online retail sales reached a new high, with more than 20% of the total retail made online, and in 2020, overall e-commerce transactions exceeded 37.2 trillion yuan, increasing from around 34.8 trillion yuan the year before (Statista, 2021). Businesses benefit from the ease of conducting business and purchasing goods online and the efficient ways of receiving goods through delivery companies such as SF Express, ZTO, YTO, Best Express, and China Post. These technological conveniences stimulate new businesses and strategies.

#### Marketing and Branding

Branding is a crucial success factor in China. Chinese consumers look for reliability when purchasing. They are very aware of counterfeit and inferior quality goods and the need to make smart purchases. There is also a preference for quality over quantity because of Miànzi, a Chinese tradition of saving face. Offering expensive brands as gifts shows their financial status, affecting how counterparts treat them. The distinct nature of Chinese culture means that business people must consider consumer perceptions and brand identity. Companies, such as Coca-Cola, who were wise enough to notice this and adjust their marketing behaviors to match this trend, have been successful and profitable.

### Implications

This article proposed a reinvention of Porters’ competitive forces model to apply the strategies used in China’s entrepreneurial endeavors. The researchers proposed the addition of three additional tools to Porter’s model. First, the model measured the role of Guanxi as a powerful tool for building lasting relationships in Chinese businesses. Second, the model highlighted technology (e-commerce and logistics) as the reason for changes, innovations, and new opportunities. Finally, the model declared marketing and branding as the key factors of success in China.

The proposed eight forces approach aims to help western firms achieve a sustainable competitive advantage and to catch up on China’s rise. This conclusion was evident in the firms’ responses. Wang and Chang’s (2009) survey of the top entrepreneurs and executives operating in China established that Porter’s five forces had little impact on business practice in China. The proposed model could be very beneficial to world business in catching up on China’s business speed or gaining advantage over competitors. It will make the model a better tool for strategic decision-making and set a foundation for other researchers engaging in similar research. In its practical field, this model will aim at accessing the profitability and attractiveness of an industry and gaining a competitive advantage over existing firms. This research was not limited to just one sector. It applies to all industries, such as education and manufacturing. Therefore, a combination of western forces and Chinese forces is optimal.

Finally, as time goes on, the eight forces may change, thereby transforming their comparative significance. Therefore, companies, researchers, or students seeking to apply this framework must verify its consistency during the application period. In addition, there is scope for the proposed forces to be explored in detail in other contexts. Finally, China’s innovation is broad and expanding, and new strategic forces will emerge to foster entrepreneurial ventures.

## Data Availability Statement

The raw data supporting the conclusions of this article will be made available by the authors, without undue reservation.

## Ethics Statement

Ethical review and approval was not required for the study on human participants in accordance with the local legislation and institutional requirements. Written informed consent from the participants was not required to participate in this study in accordance with the national legislation and the institutional requirements.

## Author Contributions

CS: conceptualization, writing—review and editing, visualization, supervision, project administration, and funding acquisition. CS and CA: methodology, investigation, resources, and writing—original draft preparation. CA: software, validation, formal analysis, and data curation. All authors have read and agreed to the published version of the manuscript.

## Conflict of Interest

The authors declare that the research was conducted in the absence of any commercial or financial relationships that could be construed as a potential conflict of interest.

## Publisher’s Note

All claims expressed in this article are solely those of the authors and do not necessarily represent those of their affiliated organizations, or those of the publisher, the editors and the reviewers. Any product that may be evaluated in this article, or claim that may be made by its manufacturer, is not guaranteed or endorsed by the publisher.

## References

[B1] Aktouf O. Chenoufi M. Holford W. D. (2005). The false expectations of Michael Porter’s strategic management framework. *Probl. Perspect. Manag.* 4 181–200.

[B2] Andriotis K. (2004). Revising Porter’s five forces model for application in the travel and tourism industry. *Entropy* 15 5154–5177.

[B3] Covin J. G. Miles M. P. (1999). Corporate entrepreneurship and the pursuit of competitive advantage. *Entrep. Theory Pract.* 23 47–63. 10.1177/104225879902300304

[B4] Dennings S. (2012). What killed Michael Porter’s monitor group? The one force that matters. Forbes, 20/11/2012.

[B5] Dess G. G. (2005). “Entrepreneurial orientation as a source of innovative strategy,” in *Innovating Strategy Process*, eds Steven J. R. Floyd W. (Oxford: Blackwell Publishing).

[B6] Dulčić Ž Gnjidić V. Alfirević N. (2012). From five competitive forces to five collaborative forces: revised view on industry structure-firm interrelationship. *Proc. Soc. Behav. Sci.* 58 1077–1084. 10.1016/j.sbspro.2012.09.1088

[B7] Frohlich M. T. Westbrook R. (2001). Arcs of integration: an international study of supply chain strategies. *J. Oper. Manag.* 19 185–200. 10.1016/S0272-6963(00)00055-3

[B8] Ginsberg A. Guth W. (1990). Corporate entrepreneurship (guest editors’ introduction). *Strat. Manag. J.* 11 5–15.

[B9] Isabelle D. A. Horak K. McKinnon S. Palumbo C. (2020). Is Porter’s five forces framework still relevant? A study of the capital/labour intensity continuum via mining and IT industries. *Technol. Innov. Manag. Rev.* 10 28–41. 10.22215/timreview/1366

[B10] Jaradat S. Almomani S. (2013). The impact of Porter Model’s five competence powers on selecting business strategy: an empirical study on Jordanian food industrial companies. *Interdiscip. J. Contemp. Res. Bus.* 5 457–470.

[B11] Johnson G. Scholes K. Whittington R. (2008). *Exploring Corporate Strategy: Text & Cases.* London: Pearson Education.

[B12] Johnson R. A. Wichern D. W. (1982). *Applied Multivariate Statistical Analysis.* Hoboken, NJ: Prentice-Hall.

[B13] Lee H. Kim M. S. Park Y. (2012). An analytic network process approach to operationalization of five forces model. *Appl. Math. Model.* 36 1783–1795. 10.1016/j.apm.2011.09.012

[B14] Love J. (2013). The end of strategy? Our faculty discusses. Kellogg Insight, 07/01/2013.

[B15] McAran D. Manwani S. (2016). “The five forces of technology adoption,” in *HCI in Business, Government, and Organizations: eCommerce and Innovation. HCIBGO. Lecture Notes in Computer Science*, Vol. 9751 eds Nah F. H. Tan C. H. (Cham: Springer). 10.1007/978-3-319-39396-4_50

[B16] Mohapatra S. (2012). I.T. and Porter’s competitive forces model and strategies. *Inform. Syst. Theory.* 9 265–281. 10.1007/978-1-4419-6108-2_14

[B17] Mugo P. (2020). Porter’s five forces influence on competitive advantage in telecommunication industry in Kenya. *Eur. J. Bus. Strat. Manag.* 5 30–49. 10.47604/ejbsm.1140

[B18] Nguyen T. (2017). *Applying Strategic Analysis in Business Strategy to Enhance Competition and Innovation: A Case Study in a Small Construction Company.* Master’s thesis. Helsinki: Arcada University of Applied Sciences.

[B19] Porter M. (2008). The five competitive forces that shape strategy. *Harv. Bus. Rev.* 86 78–93. 18271320

[B20] Scheela W. J. (2001). The entrepreneurial mindset: strategies for continuously creating opportunity in an age of uncertainty. *Acad. Manag. Exec.* 15 156–157. 10.5465/ame.2001.4251568

[B21] Shamir M. Johnson L. F. (2014). *The 5 Competitive Forces Framework in a Technology Mediated Environment. Do These Forces Still Hold in The Industry of the 21st Century?* Enschede: University of Twente.

[B22] Slater S. F. Olson E. M. A. (2002). Fresh look at industry and market analysis. *Bus. Horiz.* 45 15–22. 10.1016/S0007-6813(02)80005-2

[B23] Statista (2021). *E-commerce in China: Statistics & Facts. Ma, Y.* Available online at: https://www.statista.com/topics/1007/e-commerce-in-china (accessed September 5, 2021).

[B24] Tse E. (2010). *The China Strategy: Harnessing the Power of the World’s Fastest-Growing Economy.* New York, NY: Basic Books.

[B25] Tsui S. Wong E. Chi L. K. Tiejun W. (2017). One belt, one road: China’s strategy for a new global financial order. *Month. Rev.* 68:36. 10.14452/MR-068-08-2017-01_4

[B26] Wang W. Chang P. P. (2009). Entrepreneurship and strategy in China: why “Porter’s Five Forces” may not be. *J. Chin. Entrep.* 1 53–64. 10.1108/17561390910916886

[B27] Williams B. Onsman A. Brown T. (2010). Exploratory factor analysis: a five-step guide for novices. *J. Emerg. Prim. Health Care* 8 1–13. 10.33151/ajp.8.3.93

